# Discriminating subcortical ischemic vascular disease and Alzheimer's disease by diffusion kurtosis imaging in segregated thalamic regions

**DOI:** 10.1002/hbm.25342

**Published:** 2021-01-08

**Authors:** Min‐Chien Tu, Sheng‐Min Huang, Yen‐Hsuan Hsu, Jir‐Jei Yang, Chien‐Yuan Lin, Li‐Wei Kuo

**Affiliations:** ^1^ Department of Neurology Taichung Tzu Chi Hospital, Buddhist Tzu Chi Medical Foundation Taichung Taiwan; ^2^ Department of Neurology School of Medicine, Tzu Chi University Hualien Taiwan; ^3^ Institute of Biomedical Engineering and Nanomedicine, National Health Research Institutes Miaoli Taiwan; ^4^ Department of Psychology National Chung Cheng University Chiayi Taiwan; ^5^ Center for Innovative Research on Aging Society (CIRAS) National Chung Cheng University Chiayi Taiwan; ^6^ Department of Radiology Taichung Tzu Chi Hospital, Buddhist Tzu Chi Medical Foundation Taichung Taiwan; ^7^ GE Healthcare Taipei Taiwan; ^8^ Institute of Medical Device and Imaging, National Taiwan University College of Medicine Taipei Taiwan

**Keywords:** Alzheimer's disease, canonical discriminant analysis, dementia, diffusion kurtosis imaging, diffusion tensor imaging, subcortical ischemic vascular disease, thalamus

## Abstract

Differentiating between subcortical ischemic vascular disease (SIVD), Alzheimer's disease (AD), and normal cognition (NC) remains a challenge, and reliable neuroimaging biomarkers are needed. The current study, therefore, investigated the discriminative ability of diffusion kurtosis imaging (DKI) metrics in segregated thalamic regions and compare with diffusion tensor imaging (DTI) metrics. Twenty‐three SIVD patients, 30 AD patients, and 24 NC participants underwent brain magnetic resonance imaging. The DKI metrics including mean kurtosis (MK), axial kurtosis (*K*_axial_) and radial kurtosis (*K*_radial_) and the DTI metrics including diffusivity and fractional anisotropy (FA) were measured within the whole thalamus and segregated thalamic subregions. Strategic correlations by group, thalamo‐frontal connectivity, and canonical discriminant analysis (CDA) were used to demonstrate the discriminative ability of DKI for SIVD, AD, and NC. Whole and segregated thalamus analysis suggested that DKI metrics are less affected by white matter hyperintensities compared to DTI metrics. Segregated thalamic analysis showed that MK and *K*_radial_ were notably different between SIVD and AD/NC. The correlation analysis between *K*_axial_ and MK showed a nonsignificant relationship in SIVD group, a trend of negative relationship in AD group, and a significant positive relationship in NC group. A wider spatial distribution of thalamo‐frontal connectivity differences across groups was shown by MK compared to FA. CDA showed a discriminant power of 97.4% correct classification using all DKI metrics. Our findings support that DKI metrics could be more sensitive than DTI metrics to reflect microstructural changes within the gray matter, hence providing complementary information for currently outlined pathogenesis of SIVD and AD.

## Graphical abstract

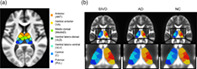

## 1 INTRODUCTION

Alzheimer's disease (AD) and vascular dementia are the first and second most common causes of dementia, respectively (Ramirez‐Gomez et al., 2017; Reed et al., 2007). Subcortical ischemic vascular disease (SIVD) represents a subtype of vascular dementia characterized by cognitive impairment and evidence of ischemic pathology confined within subcortical regions (Reed et al., 2007). Pathological evidence suggests that amyloid plaques and neurofibrillary tangles are associated with consequent cognitive deficits in AD, while microvascular and white matter damage are pathological hallmarks commonly identified in SIVD (Ramirez‐Gomez et al., 2017; Reed et al., 2007). Although AD and SIVD have distinct pathological features, differentiating between them remains a challenge due to their overlapping clinical presentations (Ramirez‐Gomez et al., 2017; Reed et al., 2007). Being labeled with distinct therapeutic and prognostic viewpoints (Ramirez‐Gomez et al., 2017; Reed et al., 2007), identifying reliable biomarkers to discriminate between SIVD and AD is important.

Neuroimaging appears to be a bottleneck on the route to diagnostic precision. Given that confluent white matter hyperintensities (WMHs) often indicate the possibility of full‐blown SIVD (Erkinjuntti et al., 2000) and that WMH load varies by dementia subtype (Tu et al., 2017), previous studies have explored the diagnostic value of diffusion tensor imaging (DTI) (Oishi, Mielke, Albert, Lyketsos, & Mori, 2011; Tu et al., 2017). Numerous DTI studies have evaluated white matter in the context of regional or whole brain analysis (Oishi et al., 2011; Tu et al., 2017). Although potentially useful, one of the technical considerations is that DTI reconstruction is based on its premise Gaussian assumption, and its quantitative metrics may not fully resolve the diffusion characteristics in complex tissue microstructures (Wheeler‐Kingshott & Cercignani, 2009). Beyond DTI, diffusion kurtosis imaging (DKI) is an advanced diffusion magnetic resonance imaging (MRI) technique which can be used to quantify intravoxel non‐Gaussian diffusion characteristics (Steven, Zhuo, & Melhem, 2014). Previous studies have demonstrated that DKI could be more sensitive in tissue characterization than DTI (Steven et al., 2014; Struyfs et al., 2015). Considering that microstructural derangements likely precede WMHs identified by conventional MRI, neuroimaging biomarkers with greater sensitivity, such as DKI metrics, are expected to provide higher clinical values in discriminating SIVD from AD.

Clinical research has associated thalamic lacunes with widespread cerebral metabolic derangements in both SIVD and AD (Schuff et al., 2003); however, the underlying tissue destruction and vascular load might be different between SIVD and AD. The thalamus mediates several essential cognitive functions, including motivation, motor control, and sensory input processing (Mai & Majtanik, 2018). As the thalamus serves as a relay station for extensive circuits connecting gray and white matter (Mai & Majtanik, 2018), it may be considered as a candidate hub to differentiate between SIVD and AD. Although the discriminative power of DKI in the white matter of patients with SIVD and AD has been reported (Oishi et al., 2011; Raja, Rosenberg, & Caprihan, 2019; Tu et al., 2017), research on mapping quantitative DKI metrics in segregated thalamic regions to differentiate between SIVD and AD is still lacking. Therefore, the aim of this study was to investigate the discriminative ability of DKI metrics for SIVD and AD compared with conventional DTI metrics. Besides, the associations between DKI metrics and cognitive measures were also explored and discussed.

## 2 MATERIALS AND METHODS

### 2.1 Participants

Twenty‐three patients with SIVD, 30 patients with AD, and 24 participants with normal cognition (NC) were enrolled. The patients with SIVD and AD shared common inclusion criteria including (a) cognitive complaints with interference in daily activities, (b) Clinical Dementia Rating (CDR) score ≥0.5 (Morris, 1993), and (c) Mini‐Mental State Examination (MMSE) score ≤26 (Shyu & Yip, 2001). SIVD and AD were diagnosed according to a previous study by Erkinjuntti et al. (2000) for SIVD and the National Institute on Aging‐Alzheimer's Association Criteria (McKhann et al., 2011), in conjunction with Hachinski Ischemic Scale assessment (Hachinski et al., 1975). The exclusion criteria were: (a) state of delirium; (b) recent stroke event within 2 weeks; (c) appearance of cortical infarcts, hemorrhages, signs of normal pressure hydrocephalus, or specific causes of white matter lesions (e.g., multiple sclerosis, sarcoidosis, brain irradiation); (d) unknown metabolic derangements contributing to cognitive impairment (e.g., abnormal levels of free T4, cortisol, folic acid, or vitamin B12); and (e) severe hearing or visual impairment. The NC participants were free from cognitive symptoms, and their MMSE scores were all >26. This study was approved by the Institutional Review Board at our hospital (#REC‐106‐09) and informed consents of all participants were well received.

### 2.2 Global cognition assessment

The MMSE is a 30‐point questionnaire (Shyu & Yip, 2001). The Cognitive Abilities Screening Instrument (CASI) is a more comprehensive assessment spanning nine cognitive domains, with a total score ranging from 0 to 100 (Lin, Wang, Liu, & Teng, 2012). Both MMSE and CASI are the tests administered to the participants, therefore the scores are determined by the participants. Higher MMSE and CASI scores represent better global cognition. The CDR assesses dementia severity through a structured interview with a reliable informant. We chose CDR sum of box for statistical analysis based on its primary nonparametric property (Morris, 1993). Given the aforementioned psychometric property, data including MMSE, CASI, and CDR sum of box were all presented in the current study to better delineate the cognitive status of the participants and their associations with neuroimaging metrics.

### 2.3 MRI protocols

All participants underwent brain MRI on a 3 T scanner (Discovery MR750, GE Medical Systems, Milwaukee, WI) with an eight‐channel phased‐array head coil. The MRI protocols included three‐dimensional T1‐weighted imaging (3D‐T1), T2 fluid‐attenuated inversion recovery imaging (T2‐FLAIR), and DKI. For 3D‐T1, a spoiled gradient echo with RF‐spoiling scheme was utilized with repetition time (TR) of 7.904 ms, echo time (TE) of 3.06 ms, inversion time (TI) of 450 ms, flip angle of 12°, matrix size (MTX) of 240 × 240 × 160 with isotropic voxel size of 1 mm^3^. Sequence parameters for T2‐FLAIR were TR of 12,000 ms, TE of 120 ms, TI of 2,200 ms, field‐of‐view (FOV) of 220 mm, MTX of 384 × 224, slice thickness (SL) of 5 mm, and 21 slices. For DKI, spin‐echo diffusion echo‐planar images were acquired with 30 diffusion gradient directions and two *b*‐values (1,000 and 2,000 s/mm^2^) along each direction. The other parameters were TR of 6 s, TE of 68 ms, FOV of 240 mm, MTX of 96 × 96, SL of 2.5 mm, and 60 slices. For each slice, a total of 65 images were acquired for DKI reconstruction, including five un‐weighted images (b0, *b* = 0 s/mm^2^) and 60 diffusion‐weighted images (DWIs with two *b*‐values along 30 diffusion gradient directions). WMHs were rated according to the Fazekas scale from T2‐FLAIR images, which semiquantifies periventricular and deep WMHs depending on the size and confluence of the lesions (Fazekas, Chawluk, Alavi, Hurtig, & Zimmerman, 1987).

### 2.4 Diffusion MRI analysis and thalamic segmentation

All image processing and registration steps were performed using AFNI software (https://afni.nimh.nih.gov) (Cox, 1996). The procedures for 3D‐T1 image co‐registration are listed step‐by‐step below. Initially, the 3D‐T1 images of each subject were skull‐removed and aligned to the MNI T1 template via 12‐parameter affine alignment. After the initial alignment, the roughly aligned T1 images were subjected to tissue extraction and the image intensity of all cerebrospinal fluid (CSF) voxels was set to zero to generate the CSF‐free 3D‐T1 images. The same process was applied to the MNI T1 template to generate the CSF‐free MNI T1 template. After this process, the CSF‐free 3D‐T1 images were nonlinearly co‐registered to the CSF‐free MNI T1 template, and this nonlinear warping transformation was applied on the roughly aligned 3D‐T1 images to generate warped 3D‐T1 images. The warped 3D‐T1 images were again nonlinearly co‐registered to the MNI T1 template to yield the final aligned 3D‐T1 images. In our study, the nonlinear registration was carried out twice. Since the ventricle sizes vary across subjects, the proposed intermediate procedure to remove the CSF voxels was carried out to achieve adequate image alignment results, particularly in thalamic regions. All of the transformation matrices, as well as warp parameters derived from the abovementioned T1 co‐registration procedures, were used for the following alignment of DWIs. DWIs were then denoised (Manjón et al., 2013) before DKI reconstruction was performed. In our study, all the diffusion data analyses were implemented using in‐house MATLAB scripts (MathWorks, MA). DKI reconstruction was performed based on the estimation approach of DKI model proposed by Tabesh, Jensen, Ardekani, and Helpern (2011). Least‐squared error estimations were performed by fitting the DKI model using the Levenberg–Marquardt algorithm. The diffusivity and kurtosis metrics along all diffusion gradient directions were derived and averaged by using diffusion data with all *b*‐values (i.e., 1,000 and 2,000 s/mm^2^). The quantitative metrics of DTI (MD: mean diffusivity; *D*_axial_: axial diffusivity; *D*_radial_: radial diffusivity; FA: fractional anisotropy) and DKI (MK: mean kurtosis; *K*_axial_: axial kurtosis; *K*_radial_: radial kurtosis) were then calculated from the DKI model.

**FIGURE 1:**
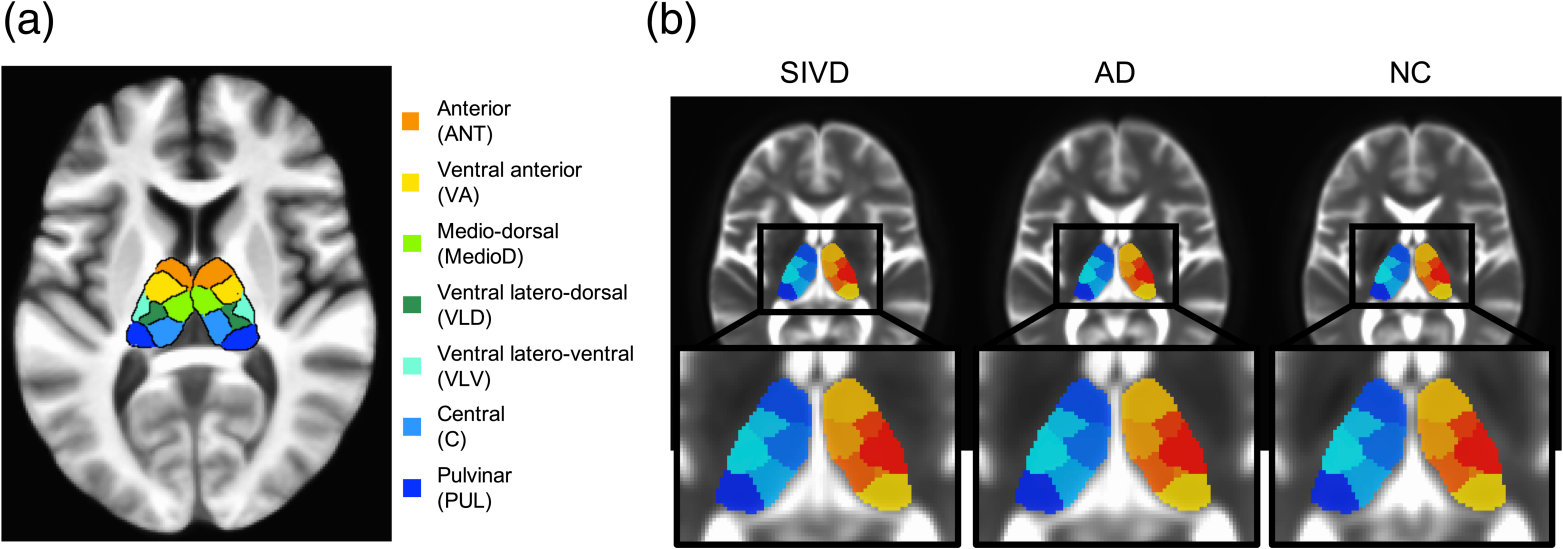
(a) Segregated thalamic subregions used in this study. (b) The final ventricle‐masked segregated thalamus atlas was overlaid onto the averaged b0 images across all three groups (SIVD, subcortical ischemic vascular disease; AD, Alzheimer's disease; NC, normal cognition)

All DKI and DTI metrics were calculated first in each subject's space. The metrics for the whole thalamus were calculated by averaging the metrics derived from the bilateral thalami [i.e., (the right thalamus + the left thalamus)/2]. For alignment of DWIs, the b0 images were first registered to each subject's own 3D‐T1 images via 12‐parameter affine alignment. The abovementioned T1‐related transformation matrix and warp parameters were applied onto the T1‐aligned b0 images to generate aligned b0 images in the MNI space. Finally, the DKI parametric maps were registered to MNI space by applying the transformations previously determined on b0 images. To reduce potential registration bias surrounding the edge of ventricular space and thalamus, a ventricle mask was generated from averaged b0 images of the SIVD group by AFNI software to avoid including unwanted ventricular voxels. A segregated thalamus atlas in MNI space (Najdenovska et al., 2018) was used for region‐of‐interest (ROI) analysis, including the pulvinar (PUL), anterior (ANT), mediodorsal (MedioD), ventral–lateral–dorsal (VLD), central (C), ventral‐anterior (VA), ventral–lateral–ventral (VLV) (Figure 1a). Representative slices of final masked thalamic subregions overlaid on averaged b0 images of the three groups are shown in Figure 1b. The segregated thalamus atlas was then used for further ROI analysis of DKI parameters. Note that the thalamus volume of an individual subject in the native space has been transformed to the thalamus volume in the MNI space, yielding no difference of thalamic volumetrics across subjects. Since the volumetrics of the transformed thalamus would not be expected to significantly affect the DKI comparison, the quantitative assessment of thalamic volumetrics was not included as covariates in the following statistical analysis.

### 2.5 Diffusion fiber tractography and thalamo‐frontal connectivity analysis

Diffusion fiber tractography was carried out using the DSI studio (http://dsi-studio.labsolver.org). Three thalamic subregions, including ANT, MedioD, and VA, and ipsilateral superior frontal gyrus were selected as seeding regions to investigate the thalamo‐frontal connectivity using a deterministic fiber tracking algorithm based on DTI orientations (Yeh, Verstynen, Wang, Fernandez‐Miranda, & Tseng, 2013). The tracking parameters included angular threshold of 30°, step size of 1 mm, and anisotropy threshold of 0.2. The fiber trajectories were smoothed by averaging the propagation direction with a given percentage of the previous direction, which was randomly selected between 0 and 95%. Tracts with a length <30 mm or >300 mm were discarded. A total of 3,000,000 seeds were placed. Fiber tracts that passed any two of the seed regions were included as fiber counts, where FA and MK values along the reconstructed fiber tracts were averaged.

### 2.6 Statistical analysis

Analysis of variance and the chi‐square test were used to compare demographics. Data from diffusion metrics were initially examined by a quality assessment process, including (a) the data of all participants were explored according to their transformed *Z* score based onto the mean/*SD* of the current dataset, (b) univariate data with *Z* score >2 or *Z* score <−2 were regarded as outliers then removed before entering variables into further statistical analysis, and (c) quantile–quantile plot for each set of data were visually examined for identifying other outlier and confirming their numeric distribution to be approximately normal. Analysis of covariance was used to examine between‐group differences by controlling for the Fazekas scale, age (Falangola et al., 2008), and education level as covariates. Between‐group differences were further examined by false discovery rate (FDR) on effect related to considering multiple comparisons (Benjamini & Hochberg, 1995). Effect size estimates were provided wherever appropriate. A partial eta‐squared (*ηp* ^2^), 0.01 ≤ *ηp* ^2^ < 0.06 denotes a small effect, 0.06 ≤ *ηp* ^2^ < 0.14 denotes a median effect, while 0.14 ≤ *ηp* ^2^ denotes a large effect (Lakens, 2013). Linear regression analysis with a stepwise regression procedure, followed by identifying significant correlations between diffusion MRI metrics and cognitive measures by partial correlation, was used to determine the best candidate to predict dementia severity and global cognition. To exclude the possible effect derived from the outliers, the Mahalanobis distance for each independent variable used in the linear regression analysis was derived. Those variables with the Mahalanobis distance/degree of freedom >2 or <−2 were regarded as outliers and then removed from the repeating linear regression analysis. Discriminant analysis was used to examine the ability of DKI to discriminate between SIVD, AD, and NC. The following validation test, in which 1,000 times of simple sampling from the biased‐corrected and accelerated percentile bootstrap after removal of the outliers, was proceeded to examine the findings derived from the original discriminant analysis. All statistical tests were performed using SPSS software version 19 (IBM, Armonk, NY). A *p*‐value <.05 was considered to be statistically significant.

## 3 RESULTS

### 3.1 Demographics

Table 1 shows the participants' demographic data. There were no significant differences in age, gender, education, symptom duration, and global cognition between SIVD and AD. The SIVD group had a higher Fazekas scale and Hachinski Ischemic Scale than the AD and NC groups (both *p* < .001). The NC group was younger than the SIVD and AD groups (both *p* < .001), and had a higher education level than the SIVD group (*p* = .023). Of 23 cases with SIVD, 12 cases (52%) were identified as having thalamic lesions such as lacunes and dilated Virchow Robin space within the thalamus by their conventional MRI scans.

**TABLE 1:** Demographic information

	SIVD	AD	NC	
	*n* = 23	*n* = 30	*n* = 24	*p*
Age (years)	74.0 (8.52)	78.3 (6.43)	65.9 (7.31)	***<*.*001*** ^a^ ^,^ ^b^ ^,^
Education (years)	7.5 (2.71)	7.9 (3.53)	10.0 (3.36)	**.*017*** ^a^
Duration (years)	2.3 (2.12)	3.1 (1.69)	0.0 (0.0)	***<*.*001*** ^a^ ^,^ ^b^ ^,^
Gender (male/female)	12/11	15/15	12/12	.984
Handedness (right/left)	22/1	29/1	24/0	.847
Hachinski ischemic scale	9.7 (2.50)	1.7 (1.33)	0.9 (0.90)	***<*.*001*** ^a^ ^,^ ^c^ ^,^
Fazekas scale periventricular white matter hyperintensities	2.0 (0.56)	1.0 (0.58)	0.5 (0.58)	***<*.*001*** ^a^ ^,^ ^b^ ^,^ ^c^ ^,,^
Deep white matter hyperintensities	2.0 (0.51)	0.7 (0.59)	0.5 (0.50)	***<*.*001*** ^a^ ^,^ ^c^ ^,^
Total	4.1 (0.92)	1.7 (0.95)	1.1 (0.85)	***<*.*001*** ^a^ ^,^ ^c^ ^,^
Clinical dementia Rating_sum of box	6.3 (4.57)	4.6 (2.64)	0.6 (0.51)	***<*.*001*** ^a^ ^,^ ^b^ ^,^
Cognitive abilities screening instrument	59.3 (19.73)	62.8 (17.36)	87.5 (4.91)	***<*.*001*** ^a^ ^,^ ^b^ ^,^
Mini‐mental state examination	18.2 (6.63)	20.9 (4.37)	27.7 (1.61)	***<*.*001*** ^a^ ^,^ ^b^ ^,^

*Notes*: Data are presented as mean (*SD*) unless otherwise stated. Comparisons were made using analysis of variance (ANOVA) and the chi‐square test where appropriate. *p* values <.05 are marked in bold italics. *p* values <.05 resulting from post‐hoc Tukey analysis are labeled as given below.

Abbreviations: AD, Alzheimer's disease; NC, normal cognition; SIVD, subcortical ischemic vascular disease.

^a^ Between SIVD and NC.

^b^ Between AD and NC.

^c^ Between SIVD and AD.

### 3.2 Assessment in bilateral thalami

Table 2 shows comparisons of averaged quantitative DTI and DKI metrics in bilateral thalami. On controlling for age and education, significant differences between SIVD and AD existed in *K*_radial_ and MK of the bilateral thalami (*p* = <.001–.006; *ηp* ^2^ = .137–.252), and *D*_axial_, *D*_radial_, and MD of the right thalamus (*p* = .005–.028; *ηp* ^2^ = .130–.142). Significant differences between SIVD and NC existed in MK of the bilateral thalami and *D*_axial_ of the right thalamus (*p* = .013–.025; *ηp* ^2^ = .130–.252). After also controlling for the Fazekas scale, the SIVD group had lower MK values within the right thalamus than the AD group (*p* = .037; *ηp* ^2^ = .092).

**TABLE 2:** Comparisons of quantitative DTI and DKI metrics

Region	Diffusion metrics				Controlling for age and education						Controlling for age, education, and the Fazekas scale					
		SIVD (*n* = 23)	AD (*n* = 30)	NC (*n* = 24)	ANCOVA			*p* (95% CI)			ANCOVA			*p* (95% CI)		
					*F*	*p*	*ηp ^2^*	SIVD vs. AD	SIVD vs. NC	AD vs. NC	*F*	*p*	*ηp ^2^*	SIVD vs. AD	SIVD vs. NC	AD vs. NC
Left thalamus	*D*_axial_	1.46 ± 0.20	1.41 ± 0.17	1.28 ± 0.16	3.030	.055	.082	.216 (−.032, .216)	.079 (−.010, .263)	1 (−.106, .174)	0.288	.751	.009	1 (−.169, .218)	1 (−.146, .261)	1 (−.107, .173)
	*D*_radial_	0.95 ± 0.20	0.88 ± 0.19	0.79 ± 0.13	3.260	**.*044***	.085	.090 (−.012, .239)	.126 (−.022, .256)	1 (−.140, .147)	0.055	.947	.002	1 (−.169, .217)	1 (−.179, .230)	1 (−.141, .144)
	MD	1.11 ± 0.19	1.05 ± 0.17	0.95 ± 0.14	3.052	.054	.082	.120 (−.018, .220)	.124 (−.020, .242)	1 (−.125, .144)	0.128	.880	.004	1 (−.153, .216)	1 (−.155, .234)	1 (−.126, .142)
	FA	0.31 ± 0.05	0.33 ± 0.05	0.32 ± 0.02	1.985	.145	.054	.151 (−.055, .006)	1 (−.044, .023)	.983 (−.021, .049)	0.541	.585	.016	1 (−.048, .047)	1 (−.035, .063)	.976 (−.021, .048)
	*K*_axial_	0.44 ± 0.03	0.44 ± 0.03	0.44 ± 0.03	.044	.957	.001	1 (−.028, .022)	1 (−.029, .029)	1 (−.027, .033)	0.031	.970	.001	1 (−.040, .040)	1 (−.040, .045)	1 (−.027, .033)
	*K*_radial_	1.05 ± 0.11	1.13 ± 0.12	1.12 ± 0.08	5.492	**.*006***	.137	**.*006 (−*.*172***, ***−*.*024)***	.170 (−.148, .017)	1 (−.053, .119)	1.259	.290	.036	.408 (−.196, .047)	1 (−.168, .084)	1 (−.054, .119)
	MK	0.86 ± 0.09	0.96 ± 0.10	0.95 ± 0.06	9.620	***<*.*001***	.223	***<*.*001 (−*.*163***, ***−*.*041)***	**.*013 (−*.*151***, ***−*.*014)***	1 (−.053, .092)	2.495	.090	.070	.087 (−.190, .009)	.298 (−.175, .033)	1 (−.053, .093)
Right thalamus	*D*_axial_	1.49 ± 0.18	1.40 ± 0.15	1.29 ± 0.19	5.093	**.*009***	.130	**.*028 (*.*011***, **.*259)***	.***025 (*.*014***, **.*287)***	1 (−.124, .156)	0.070	.933	.002	1 (−.173, .205)	1 (−.170, .228)	1 (−.124, .150)
	*D*_radial_	1.01 ± 0.19	0.90 ± 0.17	0.84 ± 0.17	5.774	**.*005***	.142	**.*005 (*.*040***, **.*282)***	.090 (−.013, .255)	1 (−.178, .099)	0.450	.640	.013	1 (−.128, .242)	1 (−.181, .210)	1 (−.179, .094)
	MD	1.16 ± 0.17	1.06 ± 0.15	0.99 ± 0.17	5.269	**.*007***	.134	**.*010 (*.*028***, **.*261)***	.074 (−.008, .248)	1 (−.156, .107)	0.237	.789	.007	1 (−.135, .222)	1 (−.171, .205)	1 (−.156, .103)
	FA	0.29 ± 0.05	0.31 ± 0.05	0.30 ± 0.02	3.059	.053	.081	.081 (−.055, .002)	1 (−.031, .032)	.155 (−.006, .060)	1.954	.150	.054	1 (−.058, 033)	1 (−.033, .062)	.157 (−.006, .060)
	*K*_axial_	0.44 ± 0.04	0.44 ± 0.04	0.45 ± 0.03	.369	.692	.011	1 (−.029, .028)	1 (−.043, .022)	1 (−.044, .024)	0.617	.543	.018	1 (−.055, .034)	1 (−.069, .027)	1 (−.044, .024)
	*K*_radial_	0.95 ± 0.10	1.05 ± 0.11	1.00 ± 0.10	7.845	**.*001***	.185	**.*001 (−*.*191***, ***−*.*045)***	.630 (−.123, .039)	.096 (−.009, .161)	3.446	**.*038***	.092	.129 (−.221, .019)	1 (−.150, .099)	.100 (−.010, .161)
	MK	0.81 ± 0.06	0.88 ± 0.08	0.87 ± 0.06	11.296	***<*.*001***	.252	***<*.*001 (−*.*132***, ***−*.*040)***	.***013 (−*.*115***, ***−*.*011)***	.925 (−.032, .078)	3.337	**.*042***	.092	**.*037 (−*.*154***, ***−*.*003)***	.259 (−.135, .023)	.937 (−.032, .078)

*Notes*: Data are presented as mean ± *SD* unless otherwise stated. Analysis of covariance (ANCOVA) was used for controlling cofactors as stated in the table. Mean/axial/radial diffusivity is presented in units of 10^−3^ mm^2^ s^−1^. *p* values <.05 are marked in bold italics. 0.01 ≤ *ηp* ^2^ < 0.06 denotes a small effect, 0.06 ≤ *ηp* ^2^ < 0.14 denotes a median effect, while 0.14 ≤ *ηp* ^2^ denotes a large effect.

Abbreviations: DTI, diffusion tensor imaging; DKI, diffusion kurtosis imaging; SIVD, subcortical ischemic vascular disease; AD, Alzheimer's disease; NC, normal cognition; *D*_axial_, axial diffusivity; *D*_radial_, radial diffusivity; MD, mean diffusivity; FA, fractional anisotropy; *K*_axial_, axial kurtosis; *K*_radial_, radial kurtosis; MK, mean kurtosis.

**FIGURE 2:**
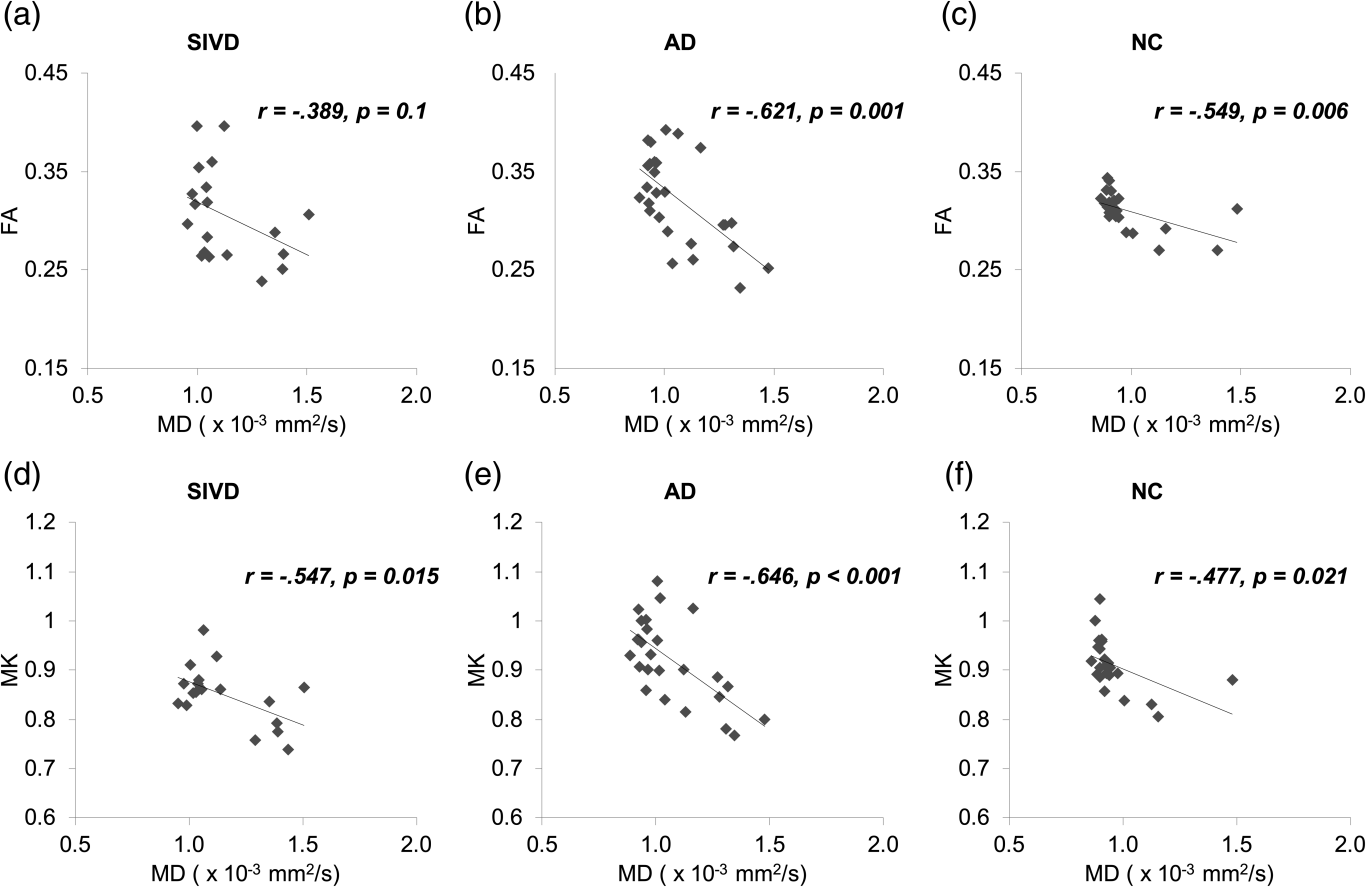
Correlation analysis between (a–c) FA and MD as well as (d–f) MK and MD across all three groups (SIVD, subcortical ischemic vascular disease; AD, Alzheimer's disease; NC, normal cognition; MD, mean diffusivity; FA, fractional anisotropy; MK, mean kurtosis)

**FIGURE 3:**
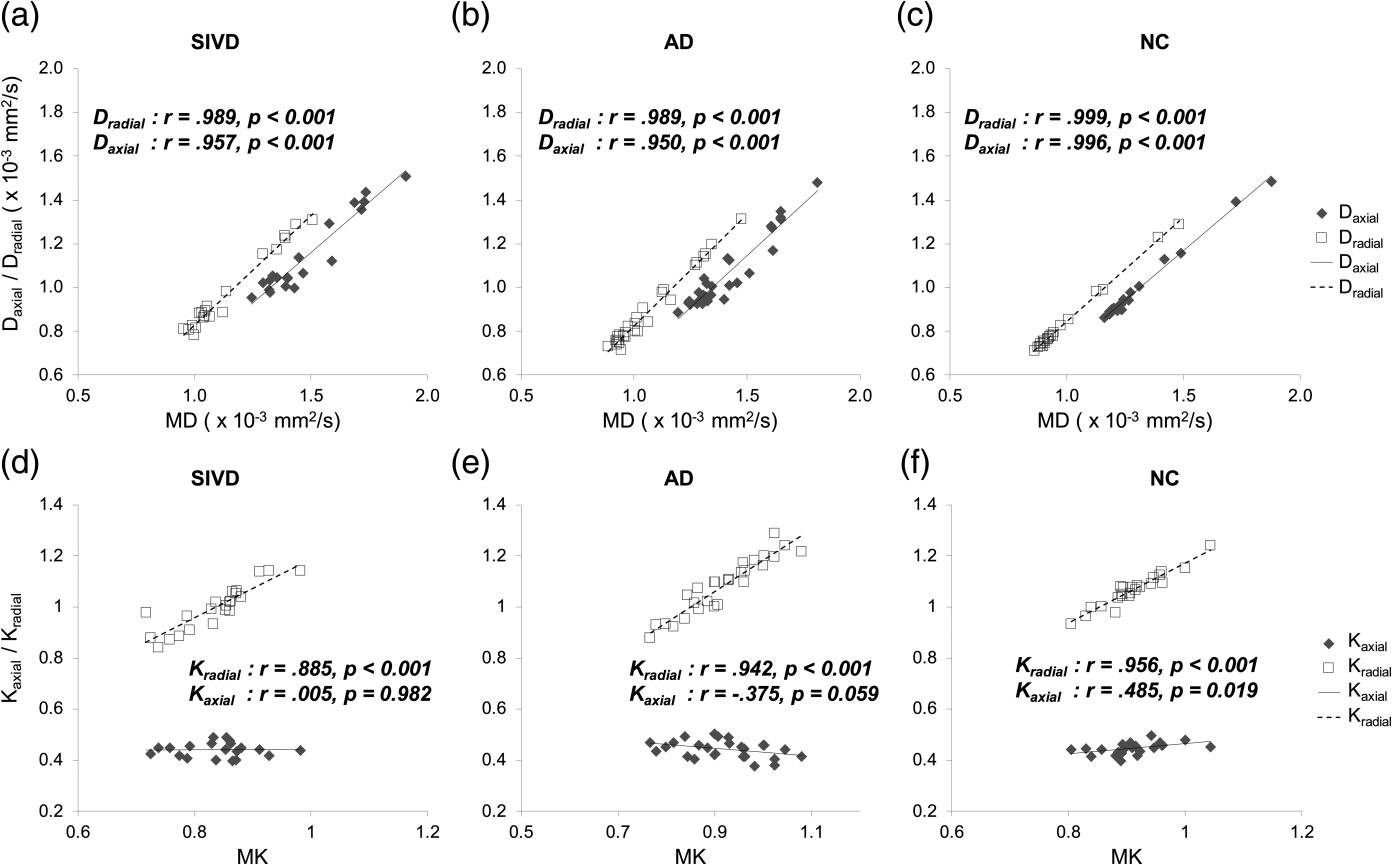
Correlation analysis among DTI and DKI metrics across the three groups. (a–c) Correlations between MD and *D*_axial_/*D*_radial_, and (d–f) Correlations between MK and *K*_axial_/*K*_radial_. DTI, diffusion tensor imaging; DKI, diffusion kurtosis imaging; SIVD, subcortical ischemic vascular disease; AD, Alzheimer's disease; NC, normal cognition; *D*_axial_, axial diffusivity; *D*_radial_, radial diffusivity; MD, mean diffusivity; FA, fractional anisotropy; *K*_axial_, axial kurtosis; *K*_radial_, radial kurtosis; MK, mean kurtosis

Figure 2 shows the correlations between MD and either of FA (a–c) and MK (d–f). With regards to MD‐FA relationships, significant negative correlations were found in AD and NC (*p* = .001–.006), compared to a nonsignificant correlation in SIVD (*p* = .100). With regards to MD‐MK relationships, significant negative correlations were found in all three groups (*p* = <.001–.021). The relationships between the mean value (i.e., MD or MK) and its axial and radial components were also examined (Figure 3). For DTI (a–c), both *D*_radial_ and *D*_axial_ showed significant positive correlations with MD in all three groups (*p* < .001). For DKI (d–f), in contrast to the significant positive correlations between *K*_radial_ and MK in all three groups (*p* < .001), the correlations between *K*_axial_ and MK varied widely among the three groups. There was a nonsignificant correlation in SIVD (*p* = .982), a trend of negative correlation in AD (*p* = .059), and a significant positive correlation in NC (*p* = .019).

### 3.3 Assessment in segregated thalamic regions

**FIGURE 4:**
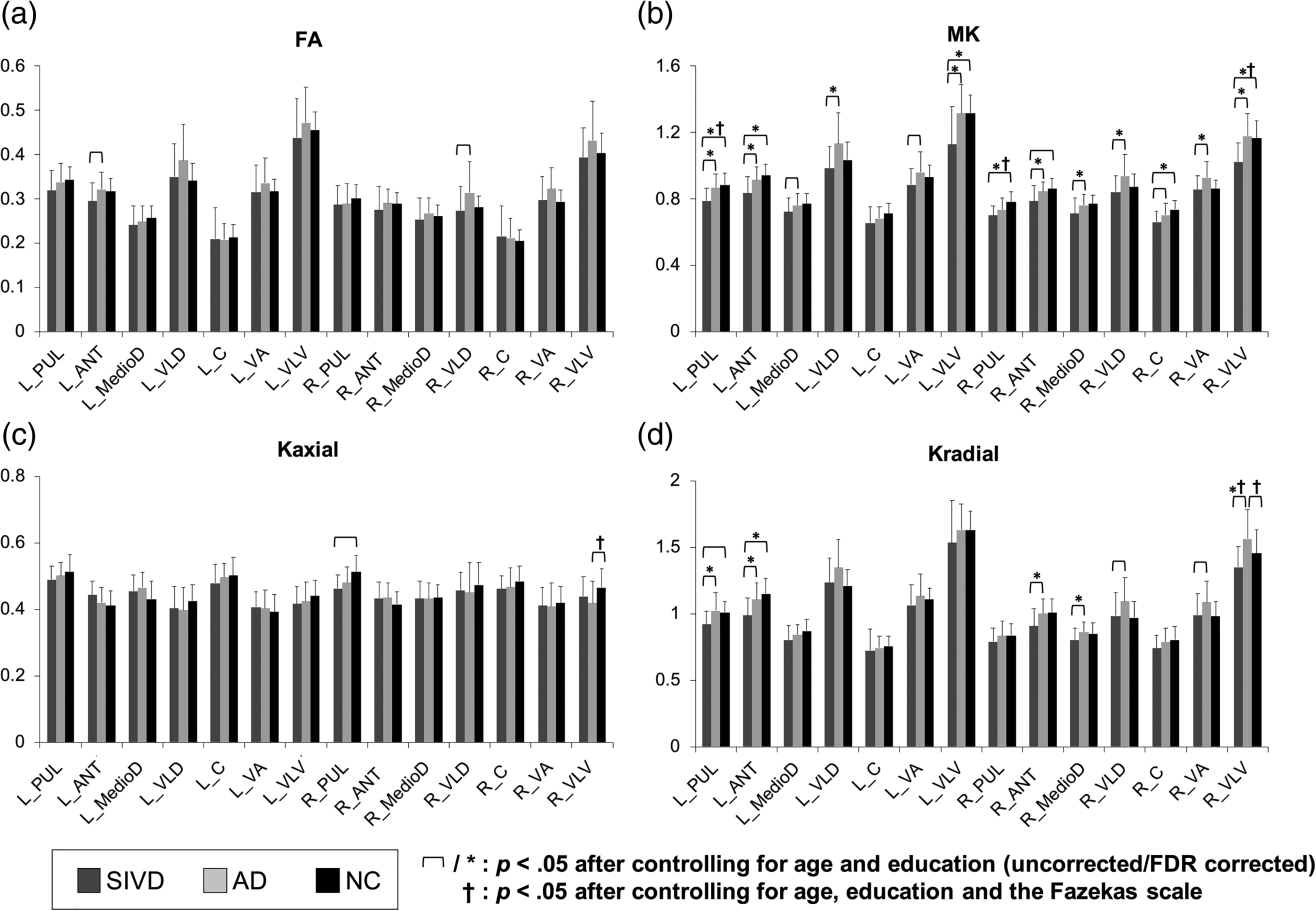
Between‐group comparisons of (a) FA, (b) MK, (c) *K*_axial_, and (d) *K*_radial_ in segregated thalamic regions. Thin brackets indicate between‐group differences with significant *p* values <.05 after controlling for age and education (FDR uncorrected). Asterisks (*) indicate between‐group differences with significant *p* values <.05 after controlling for age and education (FDR corrected). Daggers (^†^) indicate significant between‐group differences with significant *p* values <.05 noted after controlling for age, education, and the Fazekas scale. SIVD, subcortical ischemic vascular disease; AD, Alzheimer's disease; NC, normal cognition; DKI, diffusion kurtosis imaging; FA, fractional anisotropy; MK, mean kurtosis; *K*_axial_, axial kurtosis; *K*_radial_, radial kurtosis; PUL, pulvinar; ANT, anterior; MedioD, mediodorsal; VLD, ventral–lateral–dorsal; C, central; VA, ventral‐anterior; VLV, ventral–lateral–ventral; L, left; R = right

**FIGURE 5:**
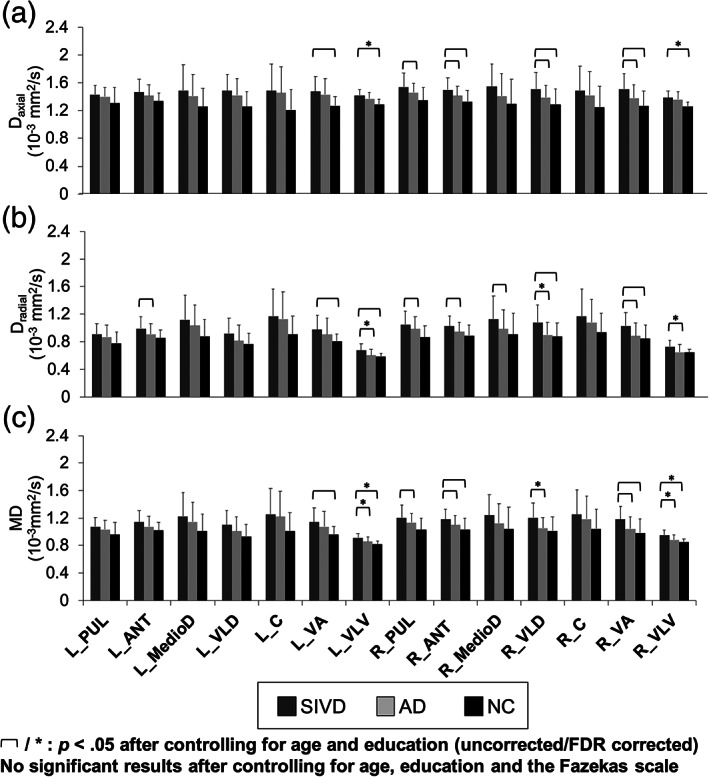
Diffusivity metrics, (a) *D*_axial_, (b) *D*_radial_, and (c) MD, in segregated thalamic regions among normal cognition (NC, *n* = 24), Alzheimer's disease (AD, *n* = 30), and subcortical ischemic vascular disease (SIVD, *n* = 23) groups. Thin brackets indicate between‐group differences with significant *p* values <.05 after controlling for age and education (FDR uncorrected). Asterisks (*) indicate between‐group differences with significant *p* values <.05 after controlling for age and education (FDR corrected). No significant between‐group differences with significant *p* values <.05 is noted after controlling for age, education, and the Fazekas scale. MD, mean diffusivity; *D*_axial_, axial diffusivity; *D*_radial_, radial diffusivity; PUL, pulvinar; ANT, anterior; MedioD, mediodorsal; VLD, ventral–lateral–dorsal; C, central; VA, ventral‐anterior; VLV, ventral–lateral–ventral; L, left; R, right

Figure 4 shows the between‐group comparisons of FA (a) and three DKI metrics (b–d) in segregated thalamic regions. No significant between‐group FA differences were noted either after controlling for age and education (FDR corrected) or also controlling for the Fazekas scale. On the contrary, DKI metrics including MK and *K*_radial_ showed more significant between‐group differences. For MK, the significant between‐group differences after controlling for age, education, and effect of multiple comparisons were found for (a) SIVD < AD in the PUL, ANT, VLD, VLV of the left side and the ANT, MedioD, VLD, VA, VLV of the right side (*p* = <.001–.006) and (b) SIVD < NC in the PUL, ANT, and VLV of the left side and the PUL, C, and VLV of the right side (*p* = .001–.014; critical value by FDR = .0179). For *K*_radial_, the significant between‐group differences after controlling for age, education, and effect of multiple comparisons were found for (a) SIVD < AD in the PUL and ANT of the left side and the ANT, MedioD, and VLV of the right side (*p* = <.001–.004) and (b) SIVD < NC in the left ANT (*p* = .002; critical value by FDR = .0083). There were only two regions (i.e., the right PUL and VLV) of *K*_axial_ showing significant differences on plain controlling for age and education (*p* = .016–.036); none has passed the FDR. On considering the Fazekas scale in addition to age and education, significant between‐group differences of MK existed in the bilateral PUL (SIVD < NC; *p* = .017–.042) and right VLV (SIVD < NC; *p* = .016), *K*_radial_ in the right VLV (SIVD < AD and AD > NC; *p* = .032–.036), and *K*_axial_ in the right VLV (AD < NC; *p* = .038). None has passed the FDR. For comparison, the assessment of diffusivity metrics is shown in Figure 5. On initial analysis, all diffusivity metrics appeared to show similar patterns of between‐group differences, that is, SIVD > AD > NC. For *D*_axial_, the significant between‐group differences after controlling for age, education, and effect of multiple comparisons were found for SIVD > NC in the bilateral VLV (*p* = .001; critical value by FDR = 0.0036). For *D*_radial_, the significant between‐group differences after controlling for age, education, and effect of multiple comparisons were found for SIVD > AD in the bilateral VLV and right VLD (*p* = <.001–.002; critical value by FDR = .0048). For MD, the significant between‐group differences after controlling for age, education, and effect of multiple comparisons were found for (a) SIVD > AD in the bilateral VLV and right VLD (*p* = <.001–.002) and (b) SIVD > NC in the bilateral VLV (*p* < .001; critical value by FDR = 0.0071). After also controlling for the Fazekas scale, none of the diffusivity metrics showed significant between‐group differences in any segregated thalamic regions.

### 3.4 Assessment of thalamo‐frontal connectivity

**FIGURE 6:**
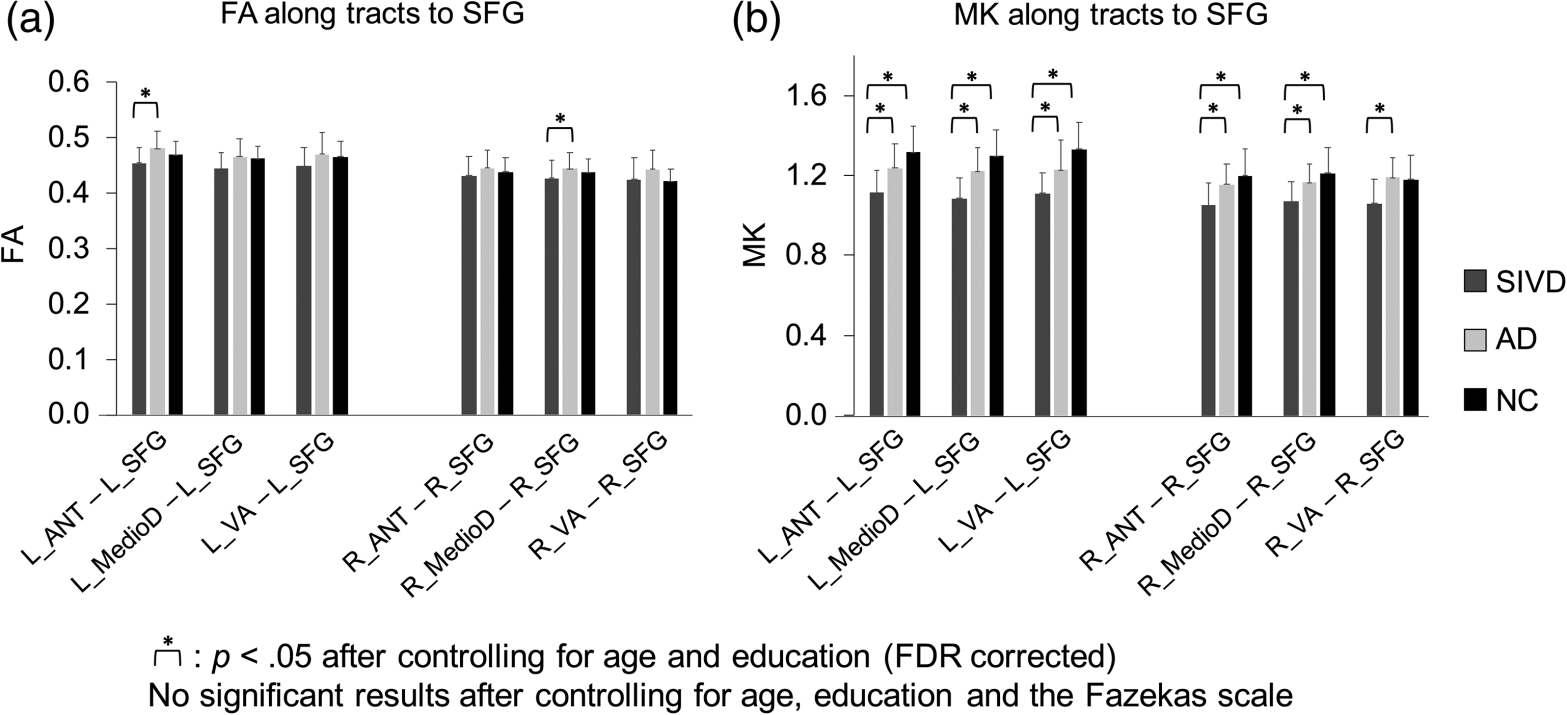
Between‐group comparisons of thalamo‐frontal connectivity. (a) FA connectivity and (b) MK connectivity. Asterisks (*) indicate between‐group differences with significant *p* values <.05 after controlling for age and education (FDR corrected). No significant between‐group differences with significant *p* values <.05 are noted after controlling for age, education, and the Fazekas scale. ANT, anterior; MedioD, mediodorsal; VA, ventral‐anterior; SFG, the superior frontal gyrus; R, right; L, left; FA, fractional anisotropy; MK, mean kurtosis

Figure 6 shows the comparisons of thalamo‐frontal connectivity among the three groups, in which the fibers connecting the superior frontal gyrus and six thalamic subregions were analyzed. The fiber tracking was conducted with variable angular threshold ranging from 30° to 60°, which revealed consistent between‐group differences in terms of the MK‐based algorithm. Therefore, we selected a dataset determined by the angular threshold of 30° for reporting here. After controlling for age and education, FA connectivity showed only two significant between‐group differences in the left ANT (SIVD < AD, *p* = .005, uncorrected) and right MedioD (SIVD < AD, *p* = .033, uncorrected), while MK connectivity showed more significant between‐group differences, including SIVD < AD in all subregions (*p* ≤ .001, uncorrected), and SIVD < NC in all thalamic subregions (*p* ≤ .001–.035, uncorrected) except for the right VA. Most aforementioned MK results passed the FDR examination, including SIVD < AD in all subregions (*p* ≤ .001) and SIVD < NC in all the left thalamic regions and the right MedioD (*p* ≤ .001–.009; critical value by FDR = .03). None of the FA results passed the FDR. No significant results were noted after also controlling for the Fazekas scale.

### 3.5 Canonical discriminant analysis

**FIGURE 7:**
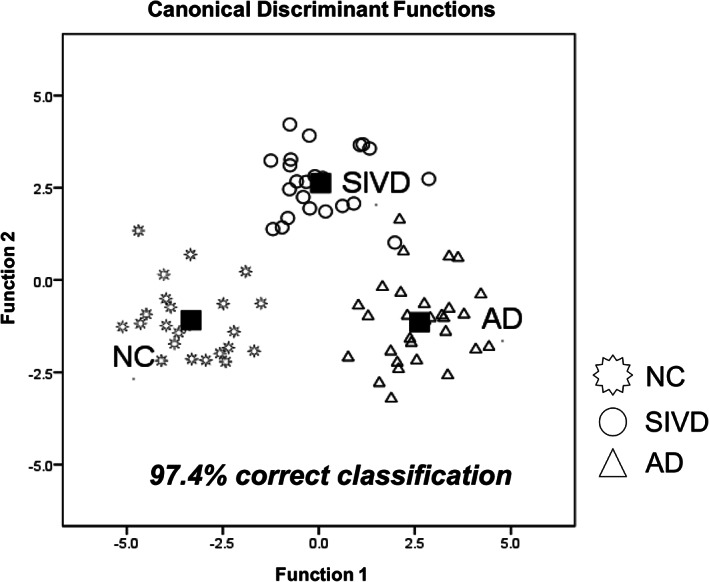
Canonical discriminant analysis using DKI metrics. SIVD, subcortical ischemic vascular disease; AD, Alzheimer's disease; NC, normal cognition

Figure 7 shows a scatter plot incorporating representative canonical discriminant functions and group centroid, in addition to other composite diffusion metrics. The dependent variable is category labeled with three levels that are mutually exclusive (i.e., 1 = SIVD, 2 = AD, and 3 = NC). The independent variables consisting of variable composite imaging metrics were entered together. The default condition was set to under equal prior probabilities. The best discriminant power was achieved by using all DKI metrics (MK, *K*_radial_, and *K*_axial_) as independent variables. Two discriminant dimensions were derived, and suggested the group means appeared to differ as Wilks' Lambda values (i.e., the proportion of overall variance in the discriminant scores unexplained by the group differences) of Function 1 to 2 and 2 were 0.033 and 0.246, respectively (both *p* ≤ .001). The canonical correlations for the first and second dimensions were 0.93 and 0.86, respectively. The Function 1/2 at group centroids are 0.048/2.626 for SIVD, 2.624/−1.142 for AD, and −3.325/−1.090 for NC. For discriminating these three groups, this method showed a discriminant power with 97.4% correct classification (*p* = .001). The discriminant power for all of the other combinations of diffusion MRI metrics is provided in Table 3. A validation test using Bootstrap was conducted after removal of the outliers defined by those with absolute *Z* value >2. The results by 1,000 times of simple sampling from the biased‐corrected and accelerated percentile bootstrap supported the original report, in which using all DKI metrics and composite metrics (MK + MD + FA) from segregated thalamic data equally achieved the best discriminant results. It was also noteworthy to point out a trend of compromised classification accuracy for those trials using single or two parameters derived from the overall thalamus data (e.g., diagnostic accuracy derived from overall MK + FA dropped from 48.1 to 45.7% by the current validation paradigm).

**TABLE 3:** Discriminant analysis by different combinations of quantitative metrics

Parameters	Classification accuracy (%)	Classification accuracy validated by bootstrap^a^ (%)
Fazekas scale^b^	71.4	–
MK^c^	57.1/77.9	54.2/77.8
MD^c^	53.2/77.9	52.1/75.3
FA^c^	50.6/70.1	47.3/68.9
(MK + MD)^c^	61.0/85.7	61.8/89.7
(MK + FA)^c^	48.1/90.9	45.7/88.6
(MK + FA)^c^	64.9/89.6	61.4/88.6
(MK + MD + FA)^c^	59.7/96.1	62.1/100.0
MK + *K*_radial_ + *K*_axial_	97.4	100.0
MK + *K*_radial_	88.3	94.4
MK + *K*_axial_	88.3	94.2
MD + *D*_radial_ + *D*_axial_	85.7	94.5
MD + *D*_radial_	81.8	78.1
MD + *D*_axial_	85.7	94.5

Abbreviations: *D*_axial_, axial diffusivity; *D*_radial_, radial diffusivity; MD, mean diffusivity; FA, fractional anisotropy; *K*_axial_, axial kurtosis; *K*_radial_, radial kurtosis; MK, mean kurtosis.

^a^ 1,000 times of simple sampling from the biased‐corrected and accelerated percentile bootstrap after removal of the outliers, defined by those with absolute value of *Z* score >2.

^b^ The classification accuracy of the Fazekas scale was derived by incorporating all components, including total score and scores of periventricular and deep white matter hyperintensities.

^c^ The classification accuracies were derived by analyzing bilateral thalami and segregated thalamic regions, respectively.

### 3.6 Linear regression analysis

Given that the best discriminant power was achieved using DKI metrics, correlations between DKI metrics and cognitive measures were examined (Table 4). After controlling for age and education, CDR sum of box showed significantly inverse correlations with (a) *K*_axial_ within the bilateral PUL, and right C, (b) *K*_radial_ within the bilateral ANT, left VLV, and right MedioD, and (c) MK within the left PUL, bilateral ANT, and left VLV. MMSE showed significantly positive correlations with (a) *K*_radial_ within the left PUL and right ANT and (b) MK within the left PUL and ANT. The aforementioned variables were first entered into preliminary stepwise linear regression models, in which the Mahalanobis distance of each independent variable was derived. The following linear regression was conducted after removal of outliers, showing that the *K*_axial_ in the right PUL (*β* = −.318, *t* = −2.927, *p* = .005) and MK in the right ANT (*β* = −.268, *t* = −2.473, *p* = .016) were the best metrics to predict dementia severity. No best predictor to predict global cognitive score was identified.

**TABLE 4:** Correlations between DKI metrics and cognition measures in all subjects (*n* = 77)

		*K*_axial_		*K*_radial_		MK	
	Subregions	CDRSOB	MMSE	CDRSOB	MMSE	CDRSOB	MMSE
Left thalamus	PUL	−.285*	.110	−.169	.243*	*−*.241*	.267*
	ANT	−.097^**†**^	.070	*−*.271*	.210	*−*.296*	.235*
	MedioD	−.029	−.057	−.019	−.008	−.079	.035
	VLD	−.113	−.084	.030	.053	−.095	.128
	C	−.077	−.034	.083	.049	−.008	.111
	VA	−.015	−.044	−.066	.095	−.082	.112
	VLV	−.194	.013	*−*.228*	.148	*−*.314**	.211
Right thalamus	PUL	−.299**	.178	.107	−.013	−.022	.073
	ANT	−.155	.019	*−*.340**	.229*	*−*.360** ^**†**^	.212
	MedioD	−.091	−.057	*−*.232*	.085	−.223	.068
	VLD	−.139	−.008	.050	−.027	−.058	.054
	C	*−*.275* ^**†**^	.057	−.069	.029	−.183	.106
	VA	−.072	.036	−.016	.027	−.063	.061
	VLV	−.205^**†**^	.146	−.010	−.052	−.092	.008

*Note*: The results were reported with partial correlation coefficients after controlling for age and education; the partial correlation coefficients with *p* values <.05 are marked in bold italics.

Abbreviations: DKI, diffusion kurtosis imaging; PUL, pulvinar; ANT, anterior; MedioD, mediodorsal; VLD, ventral‐lateral‐dorsal; C, central; VA, ventral‐anterior; VLV, ventral‐lateral‐ventral; CDRSOB, Clinical Dementia Rating sum of box; MMSE, Mini‐Mental State Examination.

*Note*: **p values <0.01. ^†^ *p* values <.05 after also controlling for the Fazekas Scale; *p values <.05.

## 4 DISCUSSION

In this study, we demonstrated that DKI metrics could more sensitively reflect the microstructural changes within the thalamus than DTI metrics. DKI can discriminate between SIVD, AD, and NC through multiple approaches, including intrinsic DKI metrics correlation, segregated thalamic assessment, thalamo‐frontal connectivity, and discriminant analysis. The utilization of DKI for delineating the thalamic microstructural changes could be a potentially useful diagnostic approach for dementia, which is consistent with the currently outlined pathogenesis of SIVD and AD (ter Telgte et al., 2018).

### 4.1 Complementary values from DKI

By theoretically resolving the intravoxel non‐Gaussianity, our results showed a greater effect size of DKI metrics than DTI metrics in differentiating the SIVD‐AD‐NC spectrum. First, MK showed more consistent patterns of correlations with MD among all three groups compared with FA. Since altered MD has been related with the cellular damage, the significant inverse relationship between MD and MK supports that decreased MK could reflect the loss of neuron/dendrites and increased extracellular space commonly associated with neurodegeneration (Steven et al., 2014). Although MK could be a more sensitive metric to characterize gray matter (Steven et al., 2014), and to detect differences between AD spectrum and controls than FA (Song, Yao, Wang, & Li, 2019; Struyfs et al., 2015), it should still be taken into account that DKI/DTI metrics could differ along with the disease course. Gong et al. (2017) have proposed DKI/DTI metric alteration is determined by the prevailing pathogenesis between microstructural neuronal loss and morphological volume reduction; elevating MD in addition to MK and probably FA decrement is hypothesized to be responsible for neuronal loss, and the opposite metric changes surrogate atrophy process that presumably dominates during the later stage.

Second, the correlation analysis between MK and its axial component showed a triphasic pattern among the three groups. Recent studies on white matter suggested that *D*_axial_ and *D*_radial_ can be considered to be surrogate markers for axon and myelin integrity, respectively (Winklewski et al., 2018), whereas *K*_axial_ and *K*_radial_ may be related to intracellular structures and cellular membrane/myelination, respectively (Hui et al., 2012). The pathological substrates corresponding to altered DKI metrics within the gray matter could be even more complicated. Given that water molecules among the SIVD pathology including gliosis, lacunes, and perivascular spaces are relatively free to diffuse, such unincorporated anisotropy of water may contribute to a considerable alteration in DTI and DKI metrics. Hui, Glenn, Helpern, and Jensen (2015) proposed a theoretical method accommodating a variety of models suitable for both white and gray matter, and suggested that the direction of kurtosis should be treated orthogonally to an axis determined by minimizing the cost function. We inferred that such a mechanism may lead to divergent MK–*K*_axial_ association among these three groups. Specifically, contrasting the positive MK–*K*_axial_ correlations as mathematically expected in NC group, lacunes, and/or perivascular spaces prevalent in SIVD could oppose considerable geometry variability in determining the axis of kurtosis. On the other hand, a trend of negative MK–*K*_axial_ in AD group could be a numeric change resulted from both deranged cellular complexity and parenchyma volume reduction.

Third, as all between‐group comparisons of DTI metrics were completely negated by considering the effects of WMHs, our results could suggest that DKI metrics are less affected by WMHs compared to conventional DTI metrics. It is also worthy to point out that only half of our SIVD subject present thalamic lesions visibly identified by their conventional brain MRI. One study using a variety of imaging metrics also identified that MK and *K*_radial_ exhibited a greater extent and degree of SIVD–AD differences within the white matter compared with conventional DTI metrics (Raja, Caprihan, Rosenberg, Rachakonda, & Calhoun, 2020). Although the thalamus is primarily made of gray matter, DKI metrics are still affected directly by the existence of lacunes and/or indirectly through Wallerian degeneration secondary to remote neuron damage. More detailed pathological evidence from postmortem studies is needed to clarify whether DKI metrics provide unique neurobiological information aside from the global state of cerebral vascular burden, as primarily indicated by the Fazekas scale (ter Telgte et al., 2018).

### 4.2 Additional values of thalamic segregation analysis

Our findings highlight the additional value of thalamic segregation analysis, as it improves the ability to categorize disease subtypes and also predicts dementia severity significantly. A consistent trend of DTI metrics (i.e., SIVD > AD > NC) in segregated thalamic regions indicates that SIVD patients may have greater cellular damage within thalamic regions than AD patients even with comparable global cognition and consideration of demographic variables. Several reports addressing DKI changes within the thalamus among dementia patients provided interesting parallels to our current work. In one study recruiting patients with AD, volume reduction of the subcortical nuclei was reported to predate DKI modifications, while kurtosis metric within thalamus was unremarkable and of no correlations with MMSE score (Wang et al., 2018). On the contrary, another report addressed the findings of MK decrement at the stage of amnestic mild cognitive impairment, yet of no significant correlation with global cognition (Gong et al., 2017). Although the discrepancy across research in this field may be due to participants' characteristics and study design, the selected regions of interests and imaging metrics could be another determinant for the results. Given that our segregated analysis shows a greater classification accuracy with scientifically sound correlation than the plain overall thalamic analysis, we are therefore convinced about the value of segregation analysis as it delivers neurobiological information of a deeper level. Together with the thalamo‐frontal connectivity evaluation, our canonical discriminant analysis indicates that a more sophisticated segregation assessment could be a pertinent solution to meet clinical needs for a higher classification rate. A similar concept is also currently proposed by Raja et al. (2020), in which a multi‐model fusion approach in evaluating major white matter tracts could provide a better diagnostic accuracy for the AD–SIVD spectrum than a uni‐model using features extracted from individual imaging metrics. Considering MK can reflect cellularity and/or structural complexity (Falangola et al., 2013), we, therefore, hypothesize that the increase in MK in the AD group may represent an effect of elective cell loss outweighed by profound volume reduction, while a state of profound cell loss with gliotic changes was associated with a decrease in MK in the SIVD group.

### 4.3 Determining neuronal substrates by linear regression analysis

Our results suggest the importance of MK changes within the ANT in determining dementia severity. Such findings corroborate the fact that the ANT contributes to reciprocal connections between the hippocampal formation and the cingulate cortex (Mai & Majtanik, 2018), hence playing a critical role in both memory and alertness. In addition, the *K*_axial_ in the PUL was another potentially useful metric to predict dementia severity, as the PUL governs sensory integration through its close interactions with the medial and lateral geniculate bodies (Mai & Majtanik, 2018).

### 4.4 Limitations

Although our results are promising, there are still some methodological concerns. First, the sample size has to be increased to validate the results, and investigations on other subtypes of dementia would be of interest. Second, although DKI metrics have been reported to be less affected by the partial volume effect than DTI metrics (Yang et al., 2013), we still need to consider its effect. In the current study, we used a ventricle mask to improve the accuracy of image registration, whereas a more sophisticated approach would be needed in further study. In addition, some other biological factors, such as blood flow from capillaries and/or glymphatic system, may contribute to the diffusion metrics. However, the corresponding fraction could be more readily identified in the context of a lower diffusion sensitivity regime (e.g., *b* ≤ 1,000), and the contributive portion within the thalamus could be relatively small (~3%) (Vieni et al., 2020). Third, the thalamic subregion template utilized here was based on local diffusion properties (Battistella et al., 2017). Although the segmentation has been shown to be similar to the histological atlas, it should be noted that the delineation of thalamus subregions varies across DWI approaches (Behrens et al., 2003; Iglesias et al., 2018; Johansen‐Berg et al., 2005). Future investigation should be made to address the efficacy in categorizing diseases via different thalamic segregation templates or segmentation approaches. Fourth, the interpretation of DKI metrics and diffusion fiber tractography should be made with caution, as changes in DKI metrics could be the result of multiple factors, including profound demyelination, crossing fibers, and residual misalignment of voxel registration (Wheeler‐Kingshott & Cercignani, 2009). Sophisticated DKI metrics such as kurtosis anisotropy, kurtosis fractional anisotropy, and generalized fractional anisotropy can be estimated to elucidate tissue with complex crossing fibers and provide more information (Glenn, Helpern, Tabesh, & Jensen, 2015). Methodologically, we examined the results of our thalamo‐frontal connectivity determined by a variable angle ranging from 30° to 60° and found that the MK performances remained consistent across the trials. Further implementation, including advanced diffusion encoding scheme and reconstruction, distortion correction approaches (e.g., eddy current correction), and sophisticated tractography algorithms, should be employed in further studies to improve the quality of DKI and strengthen our findings.

## 5 CONCLUSION

We demonstrated that DKI metrics in segregated thalamic regions could be used to classify SIVD, AD, and NC groups in terms of intrinsic metric correlation, thalamo‐frontal connectivity, and discriminant analysis. Our results show that DKI metrics could be more sensitive to characterize thalamic microstructures than conventional DTI metrics. This study highlights the potential of DKI metrics to diagnose dementia and suggests the important role of the thalamus and its connections to the frontal cortex. We believe that this study could be an inspiring starting point to investigate gray matter microstructures among different subtypes of dementia, and will be extended to a more general use in future work.

## CONFLICT OF INTEREST

The authors declare that the research was conducted in the absence of any commercial or financial relationships that could be construed as a potential conflict of interest.

## AUTHOR CONTRIBUTIONS

**Min‐Chien Tu**: study concept and design, analysis, interpretation, and drafting the manuscript. **Li‐Wei Kuo**: study concept and design, analysis, interpretation, and drafting the manuscript. **Sheng‐Min Huang**: image processing and analysis. **Yen‐Hsuan Hsu**: neuropsychological test assessment. **Jir‐Jei Yang**: neuroimaging data collection. **Chien‐Yuan Lin**: neuroimaging data collection. All authors contributed to writing the manuscript.

## ETHICS STATEMENT

This study was approved by the Institutional Review Board at our hospital (#REC‐106‐09).

## Data Availability

The derived data that support the findings of this study are available on reasonable request from the corresponding author.
